# Editorial: Community series in extreme eating behaviors—Volume II

**DOI:** 10.3389/fpsyt.2023.1200098

**Published:** 2023-04-27

**Authors:** Hubertus Himmerich, Ahmad Saedisomeolia, Ute Krügel

**Affiliations:** ^1^Department of Psychological Medicine, King's College London, London, United Kingdom; ^2^School of Human Nutrition, Faculty of Agricultural and Environmental Sciences, McGill University, Montreal, QC, Canada; ^3^Rudolf Boehm Institute of Pharmacology and Toxicology, Medical Faculty, University of Leipzig, Leipzig, Germany

**Keywords:** eating disorders, anorexia nervosa, bulimia nervosa, eating, physical health, obesity

## Introduction: recent developments in eating disorders and obesity

Currently, a significant and multi-dimensional shift in how we perceive eating disorders (EDs) and obesity is taking place.

Firstly, the perception of the spectrum of disorders associated with disordered eating has widened, leading to the addition of avoidant restrictive food intake disorder, pica and rumination disorder to the 5th edition of the Diagnostic and Statistical Manual of Mental Disorders (1), to an increased clinical and scientific interest in disordered eating in physical health problems such as cancer (2) and type 1 diabetes mellitus (3) and to a better understanding of the transdiagnostic features of body dysmorphic disorder, muscle dysmorphia and eating disorders (4).

Secondly, new genetic discoveries have opened our eyes for the metabo-psychiatric nature of eating disorders and their brain-related biological pathogeneses (5). This new perception of EDs is driving novel discoveries and developments regarding psychopharmacological treatments (6), nutritional interventions including pre- and pro-biotics (7), brain stimulation and targeted psychotherapeutic interventions to train specific brain functions such as attention (8) and impulsiveness (9). These new perspectives also help to generate an integrated view of the diagnosis and treatment of EDs and their psychological and physical co-morbidities (10). Additionally, researchers are making progress in understanding the psychological and behavioral underpinnings of obesity such as loss of control eating (11) and grazing (12).

Thirdly, the awareness of the importance of inclusiveness in EDs and obesity research is rising. For example, sexual minority groups have been identified as presenting an elevated risk for eating disorder symptoms and behaviors (13), and it has been noticed that people from ethnic minority backgrounds often experience greater difficulties in accessing support for an ED or other mental health problems (14).

Finally, there is a growing body of evidence that indicates the potential benefit of creative and receptive arts therapies in EDs and related mental health problems. For example, the therapeutic application of music such as listening to classical music, singing in a group and songwriting have been reported to be helpful in EDs (15) and in their most frequent co-morbidities like personality disorders (16) and obsessive-compulsive disorder (17).

## Content of the second volume of “Extreme Eating Behaviors”

Because of these exciting developments in the field of EDs and obesity and the success of our initial Research Topic on “Extreme Eating Behaviors” (18) published in 2019, we were encouraged to issue a second volume on this Research Topic which covers brain research, psychological and physical aspects of eating behaviors, EDs and obesity, eating-related traits of specific population groups, and novel treatments for EDs.

Armon et al. investigated differences in brain activation in response to sweet and sour tastes in female study participants with and without bulimia nervosa and found differences in several brain regions which may be central to perception and processing of taste, including the operculum, the anterior cingulate cortex, the midbrain, and the cerebellum.

Munguía et al., Hussenoeder et al., Staníková et al., and Saure et al. investigated the relationship between physical and psychological risk factors of EDs and obesity such as thyroid hormone levels, food addiction, anxiety, food preferences, sensory processing, and autistic traits. Interesting findings from their studies include the association between sensory sensitivity and food addiction on the one hand and anxious symptoms and problems with specific food groups on the other hand as well as the potential involvement of thyroid hormone signaling in food preferences and the development of obesity.

Two articles addressed the connections between EDs and physical health problems: Riedlinger et al. examined gastrointestinal complaints in patients with anorexia nervosa, and Paszynska et al. analyzed that there is a high risk of dental caries and erosive tooth wear in children and adolescents with anorexia nervosa.

The articles by Gulec et al. and Stein et al. cover eating-related psychopathological and behavioral aspects in specific minority groups, namely a lesbian, gay, bisexual, transgender, intersexual, and queer (LGBTIQ) sample in Turkey and a sample of Jewish ultra-orthodox religious men. Stein et al. found that Jewish ultra-orthodox religious males with obsessive-compulsive disorder might be at risk for severe undernutrition because their rigid observance of Jewish everyday laws might interfere with their eating. As in this group of patients, the loss of weight did not result from body image concerns, Stein et al. argue that these patients should rather be diagnosed with restricting type ED than with anorexia nervosa. However, the authors themselves indicate that their diagnostic opinion is debatable. For clarity, we would like to inform the readers that body image disturbance is not mandatory for the diagnosis of anorexia nervosa according to DSM-5 (1). A lack of insight into the seriousness of the disease together with a significantly low body weight and the fear of weight gain, for example, would suffice (1).

From a historic point of view, we would like to mention that the role of religion or religiousness has been discussed as a pathophysiological, maintaining, protective, diagnostic, or therapeutic factor of EDs from the beginning of academic psychiatry (19–21) until today (22).

Regarding novel treatments, Flynn et al. published a protocol for an innovative study that allows to test whether concurrent self-administered transcranial direct current stimulation (tDCS) and attention bias modification training might be suitable to improve symptoms of binge eating disorder.

## Expression of gratitude and good wishes

We are thankful to all the authors mentioned above who submitted excellent manuscripts for this Research Topic. We also thank our reviewers who gave useful feedback and thus helped to improve the manuscripts and the second volume in its entirety.

Even though a single Research Topic cannot cover all the facets of EDs and of obesity research, we hope that this article Research Topic is making a decent contribution to the field. In this sense, we wish to attract attention to some so far underestimated disease aspects with this second volume of “Extreme Eating Behaviors”.

## Author contributions

All authors listed have made a substantial, direct, and intellectual contribution to the work and approved it for publication.
